# Feature-specific quantile normalization and feature-specific mean–variance normalization deliver robust bi-directional classification and feature selection performance between microarray and RNAseq data

**DOI:** 10.1186/s12859-024-05759-w

**Published:** 2024-03-29

**Authors:** Daniel Skubleny, Sunita Ghosh, Jennifer Spratlin, Daniel E. Schiller, Gina R. Rayat

**Affiliations:** 1 Department of Surgery, Faculty of Medicine and Dentistry, University of Alberta, Edmonton, AB T6G 2R3 Canada; 2 Department of Oncology, Faculty of Medicine and Dentistry, University of Alberta, Edmonton, AB T6G 2R3 Canada; 3 Department of Mathematical and Statistical Sciences, Faculty of Science, University of Alberta, Edmonton, AB T6G 2R3 Canada

**Keywords:** Cross-platform normalization, Molecular classification, Microarray, RNAseq, Feature selection, Quantile normalization, Mean, Variance, FSQN, FSMVN

## Abstract

**Background:**

Cross-platform normalization seeks to minimize technological bias between microarray and RNAseq whole-transcriptome data. Incorporating multiple gene expression platforms permits external validation of experimental findings, and augments training sets for machine learning models. Here, we compare the performance of Feature Specific Quantile Normalization (FSQN) to a previously used but unvalidated and uncharacterized method we label as Feature Specific Mean Variance Normalization (FSMVN). We evaluate the performance of these methods for bidirectional normalization in the context of nested feature selection.

**Results:**

FSQN and FSMVN provided clinically equivalent bidirectional model performance with and without feature selection for colon CMS and breast PAM50 classification. Using principal component analysis, we determine that these methods eliminate batch effects related to technological platforms. Without feature selection, no statistical difference was identified between the performance of FSQN and FSMVN of cross-platform data compared to within-platform distributions. Under optimal feature selection conditions, balanced accuracy was FSQN and FSMVN were statistically equivalent to the within-platform distribution performance in multivariable linear regression analysis. FSQN and FSMVN also provided similar performance to within-platform distributions as the number of selected genes used to create models decreases.

**Conclusions:**

In the context of generating supervised machine learning classifiers for molecular subtypes, FSQN and FSMVN are equally effective. Under optimal modeling conditions, FSQN and FSMVN provide equivalent model accuracy performance on cross-platform normalization data compared to within-platform data. Using cross-platform data should still be approached with caution as subtle performance differences may exist depending on the classification problem, training, and testing distributions.

**Supplementary Information:**

The online version contains supplementary material available at 10.1186/s12859-024-05759-w.

## Introduction

Molecular classification using gene expression data provides a robust framework to research, treat, and classify human disease [1–3]. In cancer, molecular classification has delivered valuable insight into tumour heterogeneity, and disease etiology, progression, and prognosis [4–6]. Open data-sharing policies have created numerous public online compendiums to access and use gene expression data for research purposes.

Whole transcriptome gene expression is commonly measured using either microarray or RNA-sequencing (RNAseq) technology. Although both methods generate gene-level expression data, the underlying technology used to determine expression levels is inherently different [7]. Thus, comparing or combining experiments from separate technological platforms is a known problem. It is desirable to use multiple platforms of gene expression data to explore human disease because it allows external validation of experimental findings, increases sample sizes for clinical outcomes data, and augments training sets for machine learning models.

Normalizing data between gene expression platforms is commonly referred to as cross-platform normalization. The purpose of normalization is to eliminate differences in samples due to technological differences while maintaining biologically relevant characteristics. Methods that insufficiently account for technological differences or conversely methods that eliminate biological signals introduce confusion and bias.

The idea of matching the specific distribution of each gene to a reference target distribution is a well-known, but poorly characterized method. For example, in 2003 Wright et al. used the principle of feature-specific distribution matching of the *mean* and *variance* of each gene to compare the measurement of lymphoma specimens from two separate microarray probe-based technologies [8]. They claimed that this method removed systematic measurement differences between the two platforms. This method was never specifically named, and no formal validation study has been performed. However, this method has been used to learn molecular subtypes on external datasets in numerous high-impact publications such as those published in Nature Communications [9], and Clinical Cancer Research [10]. The specific mathematical calculation used in this instance has not formally been provided and to our knowledge, no software-related code publicly exists for replication purposes. In the present study, we formally identify this method as Feature Specific Mean Variance Normalization (FSMVN).

**Fig. 1:**
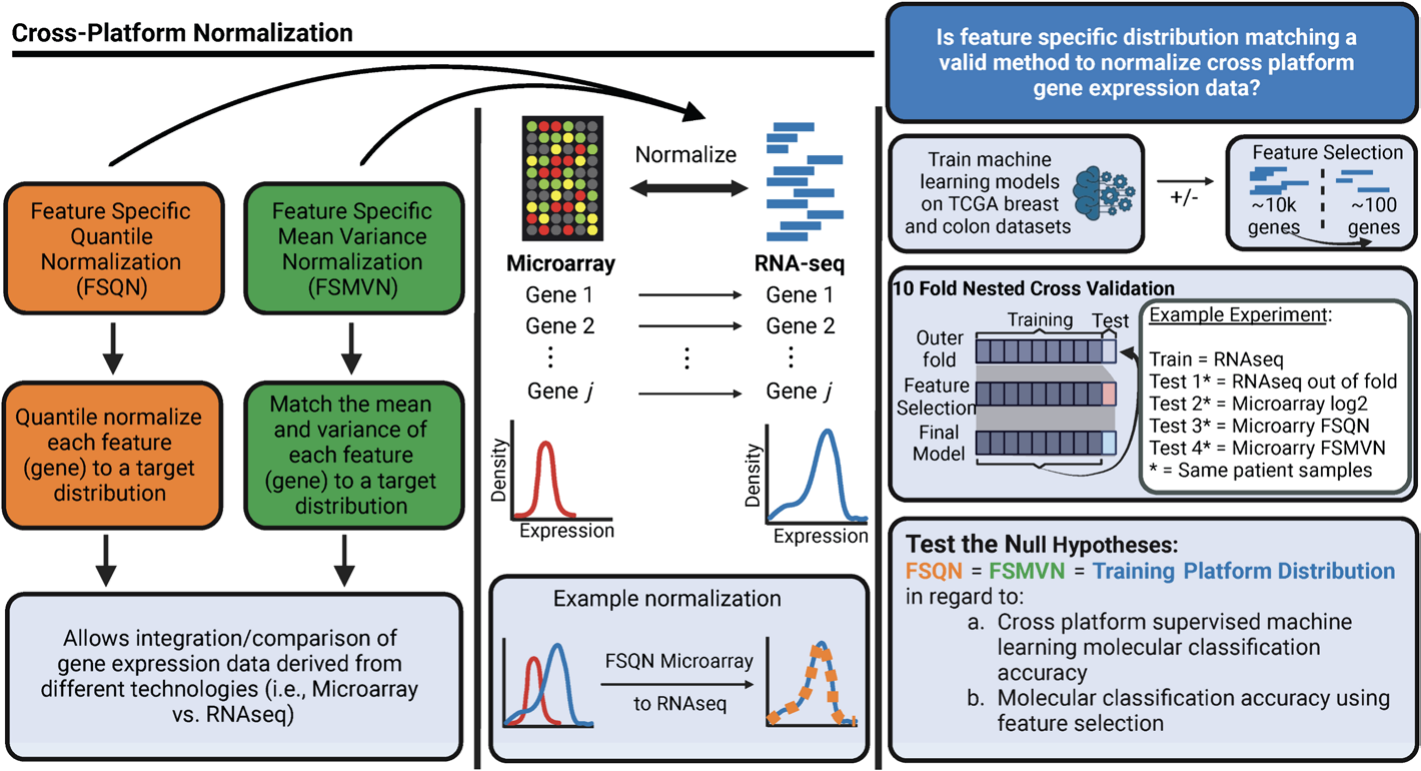
Study overview. Feature Specific Quantile Normalization and Feature Specific Mean Variance Normalization are two methods of cross-platform normalization that allow integration and/or comparison of microarray and RNAseq data. These methods use either quantile normalization or mean and variance matching at a gene-specific level to match gene expression data to a target distribution. These methods are bidirectional, in that microarray data can match the RNAseq distribution and vice versa. Here, we evaluate the validity of using FSQN and/or FSMVN in the context of supervised machine learning classifiers for molecular subtyping using cross-platform data. We compare the model accuracy to the unnormalized log_2_ gene expression data of the target/training distribution. We also evaluate whether FSQN and/or FSMVN is a valid method in the context of feature selection

In 2018, Franks et al. proposed using Feature Specific Quantile Normalization, which performs quantile normalization at the individual gene level [11] (see Fig. 1). In this study, FSQN provided superior classification performance compared to distribution level quantile normalization [12], training distribution matching [13], and non-paranormal transformation [14]. However, the utility of FSQN was only demonstrated in the unidirectional manner of transforming RNAseq to microarray data distributions. In 2023, Foltz et al. published an independent study also demonstrating the efficacy of FSQN [15]. Although not specifically referenced as FSQN, this study used the identical R function as the Franks et al. study. One important finding was that FSQN and other cross-platform normalization methods are able to normalize data in a bidirectional manner (i.e., RNAseq to microarray *or* microarray to RNAseq). However, interpretation of these data in some common experimental circumstances is limited because they used a combination of microarray and RNAseq data in the training and testing sets as opposed to pure platform-independent data.

In this study, we investigate the utility of FSQN compared to FSMVN in the context of supervised machine learning classification performance for molecular subtypes. First, we test if these methods are capable of bi-directional normalization, defined as normalization using exclusively microarray *or* RNAseq as a target distribution. Next, we explore the efficacy of these methods when using feature selection techniques. This work is novel because it is (1) the first formal analysis of FSMVN performance using gene expression data tested with both microarray and RNAseq data on the same patients, (2) the first analysis of FSQN and FSMVN using only RNAseq data as a target distribution for microarray cross-platform normalization (i.e. previous studies used mixed data as a target distribution or only normalized RNAseq to microarray data), and (3) the first to evaluate the effect of feature selection/number of selected features on cross-platform normalization performance (i.e. previous studies investigated the effect of sample size used for training).

## Methods

### Study design

Gold-standard comparison for cross-platform normalization uses data with separate technological gene expression measurements performed on the same biological sample/patient. These include The Cancer Genome Atlas (TCGA) breast (BRCA) [16] and TCGA colon adenocarcinoma (COAD) [17] data measured using both Agilent microarray and Illumina RNAseq platforms. Given that gene expression measurements are performed within the same study and on the same patient samples we assume that differences in gene-level expression are predominately due to technological platform-specific differences.

This study assesses the bidirectional performance of FSQN and FSMVN. Supervised machine learning models were created on a training distribution consisting entirely of microarray or RNAseq data. The performance of this model was then assessed in unseen, holdout test folds derived from nested cross-validation consisting of either: 1. data from same origin/platform of the training distribution (positive control); 2. data from different origin/platform that is simply log_2_ transformed (negative control); 3. data from different origin/platform that is normalized to match the training distribution using FSQN; and 4. data from different origin/platform that is normalized to match the training distribution using FSMVN.

This study also assesses whether FSQN and FSMVN are valid in the setting of feature selection. Feature selection refers to decreasing the number of features/genes using some form of criteria that is typically derived from statistical models or expert knowledge. Feature selection may enhance model performance by eliminating noise and aims to alleviate the curse of dimensionality [18]. We independently assess model performance with and without feature selection. We also directly compare the performance of feature selection versus no feature selection in multivariable models. See below for details about our feature selection methods and design.

In all cases, control and experimental groups were trained and tested on the exact same patient samples. We used identical nested cross-validation folds for each experimental procedure. Our primary outcome metric was balanced accuracy, which accounts for imbalanced class data. We also assessed the mean absolute scaled error (MASE) for gene expression matrices between log_2_, FSQN and FSMVN method to assess which method best approximates the native reference distribution gene expression values. Using these methods, we test the null hypothesis that FSQN, FSMVN, and the reference training platform distribution achieve equal model performance.

### Dataset descriptions and processing

Whole transcriptome RNAseq data was retrieved from cbioportal for TCGA breast (BRCA) and colon adenocarcinoma (COAD) experiments [19]. Next-generation sequencing data was retrieved as RNA-Seq by Expectation–Maximization (RSEM) counts and already batch normalized [20]. Data was log2(RSEM count +1) transformed. Genes with mean log2 expression < 1 were removed. EntrezIDs were mapped to gene symbols with org.Hs.eg.db using AnnotationDBI [21].

Level 3 (gene-level) Agilent custom 244 K whole genome microarray data was retrieved from Genomic Data Commons [22]. Data was retrieved as log2 loess normalized with annotated gene symbols. This data was previously known to contain no serious batch effects [16]. There was 0.18% missing data among 540 genes, which was imputed using the k-nearest neighbours algorithm.

For all datasets, we performed exploratory analysis using Bland–Altman plots, qqplots, boxplots, and probability density functions to confirm appropriate normalization. For each data set, tumour adjacent normal samples were removed. Molecular subtypes including PAM50 for BRCA [23] and Consensus Molecular Subtypes (CMS) [24] for COAD were included. For BRCA, the normal-like subtype samples were removed due to low class prevalence for the learning algorithms. Only common samples measured on Agilent and Illumina platforms with molecular subtype labels were included. Only annotated genes that were present in both RNAseq and Agilent expression data were included. Following these procedures, the final gene expression matrix for BRCA and COAD was 431 patients × 12,638 genes and 187 patients × 13,362 genes, respectively.

### Cross-platform normalization

Normalization between platforms was performed using Feature Specific Quantile Normalization and Feature Specific Mean Variance Normalization. The mathematical explanation of FSQN was previously characterized [11]. FSQN was implemented in R using the FSQN package, which utilizes the normalize.quantiles.use.target function from the preprocessCore package.

FSMVN was performed by matching the mean and variance of the test distribution to a specified target distribution for each gene/feature, respectively. Let ${{\varvec{X}}}_{i,j}$ represent a test gene matrix and ${{\varvec{Y}}}_{i,j}$ a target gene matrix, where $i$ rows represent biological samples and $j$ columns represent genes. Note, FSMVN requires ${{\varvec{X}}}_{j}= {{\varvec{Y}}}_{j}$ (i.e., identical gene features). We then applied FSMVN to normalize ${{\varvec{X}}}_{i,j}$ to${{\varvec{X}}}_{i,j}^{N}$, where N denotes a normalized matrix to the target distribution. For each feature$*,j$, where$* =\left[{x}_{1},\dots ,{x}_{i}\right]{ }_{j} \in {{\varvec{X}}}_{i,j}$, we standardize to a mean of zero and standard deviation of 1, transform the feature to the standard deviation of ${s}_{*,j }\in {{\varvec{Y}}}_{i,j}$ then add the mean${\overline{y} }_{*,j}$. Thus, for the first feature in a matrix ${x}_{i,1}\in {{\varvec{X}}}_{i,j}$, FSMVN is calculated as:

$${x}_{i,1}^{N}= \frac{{x}_{i,1}- {\overline{x} }_{*,1}}{{s}_{{x}_{*,1}}} {s}_{{\overline{y} }_{*,1}}+ {\overline{y} }_{*,1}$$

To achieve ${{\varvec{X}}}_{i,j}^{N}$ FSMVN is calculated as above for each feature $\left[{x}_{1},\dots ,{x}_{j}\right] \in {{\varvec{X}}}_{i,j}$. A FSMVN R function is provided in the supplement (Additional File 1: Feature Specific Quantile Normalization R Function).

### Machine learning models and feature selection

Supervised machine learning classifiers were created using caret in R [25]. Molecular subtypes were used as the supervised labels and genes as features. Models were trained and tested using nested stratified tenfold cross-validation (CV) [26]. Stratification of supervised labels ensured a consistent proportion of classes were present in each training and testing fold [27]. Support Vector Machine (SVM) models, implemented as ‘*svmLinear2*’ [28], and glmnet [29] models were used for feature selection and classification models. Model selection with hyperparameter optimization occurred within the middle layer of nested tenfold CV. The innermost layer was used to train feature-selected models that were subsequently tested on the holdout CV fold in the outermost layer.

Default tuning parameters were used except for glmnet models in the outermost loop. Here, glmnet was implemented as Least Absolute Shrinkage and Selection Operator (LASSO) regression (i.e., α = 1) so that features could be selected based on the minimum mean cross-validated error using the glmnet package. For SVM, the top features for each molecular subtype class were selected as defined by variable importance in caret. The number of features selected was the mean number of features selected by LASSO regression in each experimental condition. Duplicate selected genes were removed. Of note, when assessing feature selection performance, FSQN and FSMVN were performed on unseen data with a reduced number of features *after* feature selection on the full training distribution.

### Feature selection experiment

To assess whether FSQN or FSMVN performance is affected by the number of features selected we evaluated the performance of models using pre-specified feature selection. We used SVM to select approximately 10,000, 5000, 500, 100, 50, 25, or 10 features and assessed model performance.

### Statistical analysis

Statistical analyses were completed using R version 4.2.3. Summary statistics were calculated to describe the mean and empiric 95% bootstrapped confidence intervals using 1,000 bootstraps. Differences between groups were assessed using Dunn’s test (i.e., Kruskal–Wallis with multiple comparisons). *P*-values were adjusted using the Holm method to control the Type 1 error rate due to multiple comparisons. Statistical significance was defined at alpha = 0.05. Principal Component Analysis (PCA) was performed using Singular Value Decomposition in R. Multiple linear regression was performed using default parameters to summarize the overall effects of experimental conditions. Pairwise comparisons of the estimated marginal means for normalization methods variables were performed using the eemeans package. P-values for these pairwise comparisons were corrected using the Holm method. Figures were generated using ggplot2 and ggpubr packages [30].

## Results

### FSQN and FSMVN normalize distributions and eliminates platform related batch effects

**Fig. 2:**
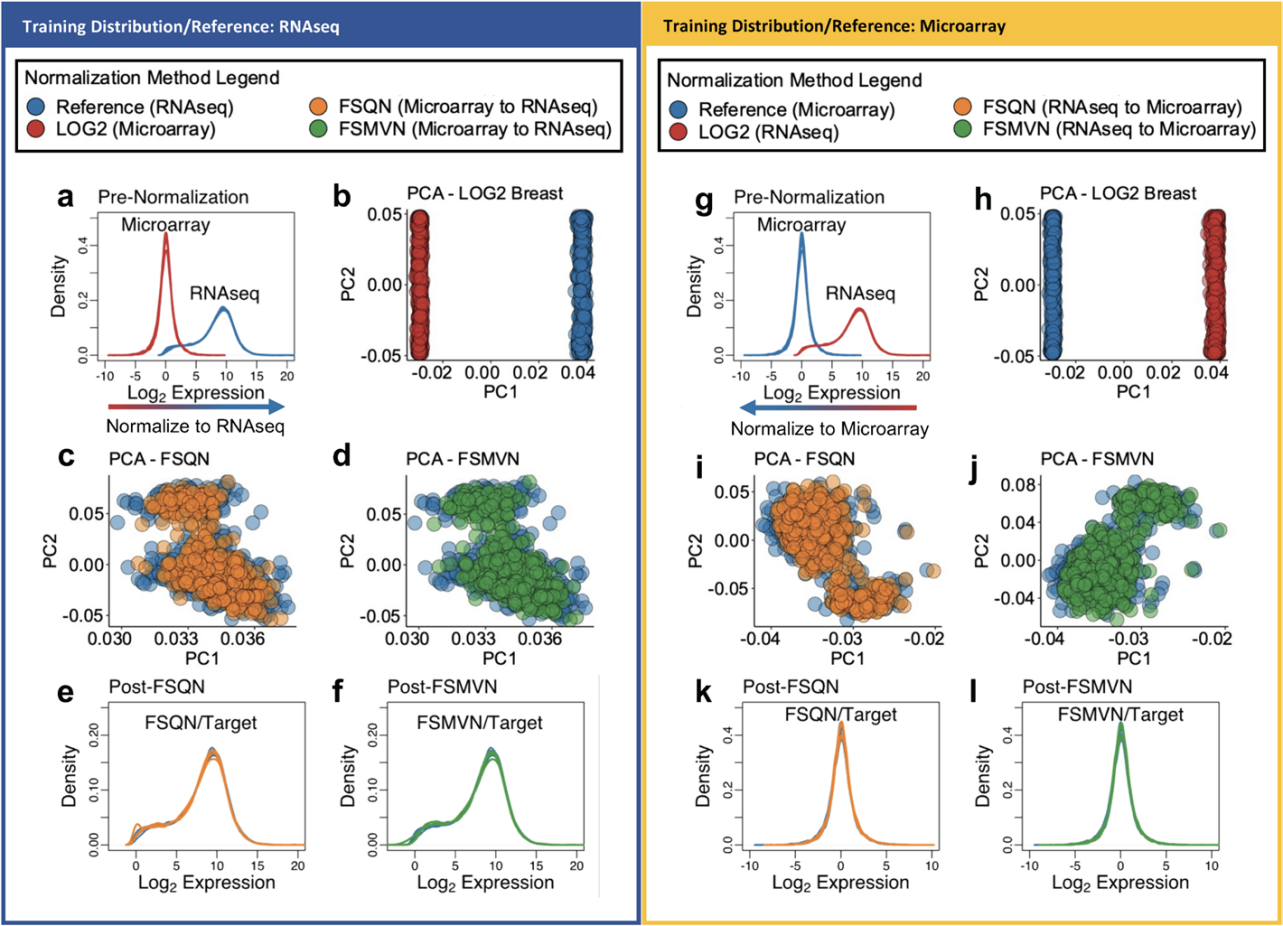
Effect of feature specific normalization methods on test and training breast distributions. Left block (Dark Blue): Normalization using RNAseq data as training distribution. Right block (Gold): Normalization using microarray data as training distribution. Colour legends for each block are provided. **a**, **g**. Probability density functions of log_2_ microarray and RNAseq data prior to feature specific normalization. **b**, **h** Principal Component Analysis (PCA) plots of log_2_ microarray data and log_2_ RNAseq data. The first (PC1) and second (PC2) principal components are projected on the x-axis and y-axis, respectively. **c**, **i** PCA plot of the first two principal components of gene expression data after feature specific quantile normalization (orange) to the respective training distribution (blue) demonstrates limited variation between gene expression platforms after FSQN. **d**, **j**. PCA plot of the first two principal components of gene expression data after feature specific mean–variance normalization (green) to training distribution (blue) demonstrates limited variation between gene expression platforms after FSMVN. **e**, **k**. The probability density function of gene expression data after FSQN demonstrates the shift of the test distribution (orange) to match the training distribution (blue). **f**, **l**. The probability density function of gene expression data after FSMVN demonstrates the shift of the test distribution (green) to match the training distribution (blue)

The baseline distribution of log_2_ transformed gene expression data for microarray and RNAseq BRCA data is illustrated in Fig. 2a and g. The greatest source of variation observed in the first principal component (x-axis) is represented by the separate technological platforms (Fig. 2b and h). The second greatest source of variation is represented by the patient samples observed along the second principal component (y-axis).

Following FSQN and FSMVN, the batch effects between technological platforms observed in Fig. 2b and h were eliminated regardless of the training distribution used (Fig. 2c, d, i, and j). Furthermore, we observed that FSQN and FSMVN projected nearly identical PCA plots within their respective training distributions. In Fig. 2e and f, the near complete overlap of probability density functions is achieved after FSQN or FSMVN of microarray to RNAseq data. Likewise, in Fig. 2k and l, we again observe a meaningful and near complete shift of the RNAseq probability density function to match the training microarray distribution. These findings in BRCA data are replicated in COAD data (Additional file 1: Fig. S1).

### FSQN and FSMVN deliver equivalent balanced accuracy without feature selection

**Fig. 3:**
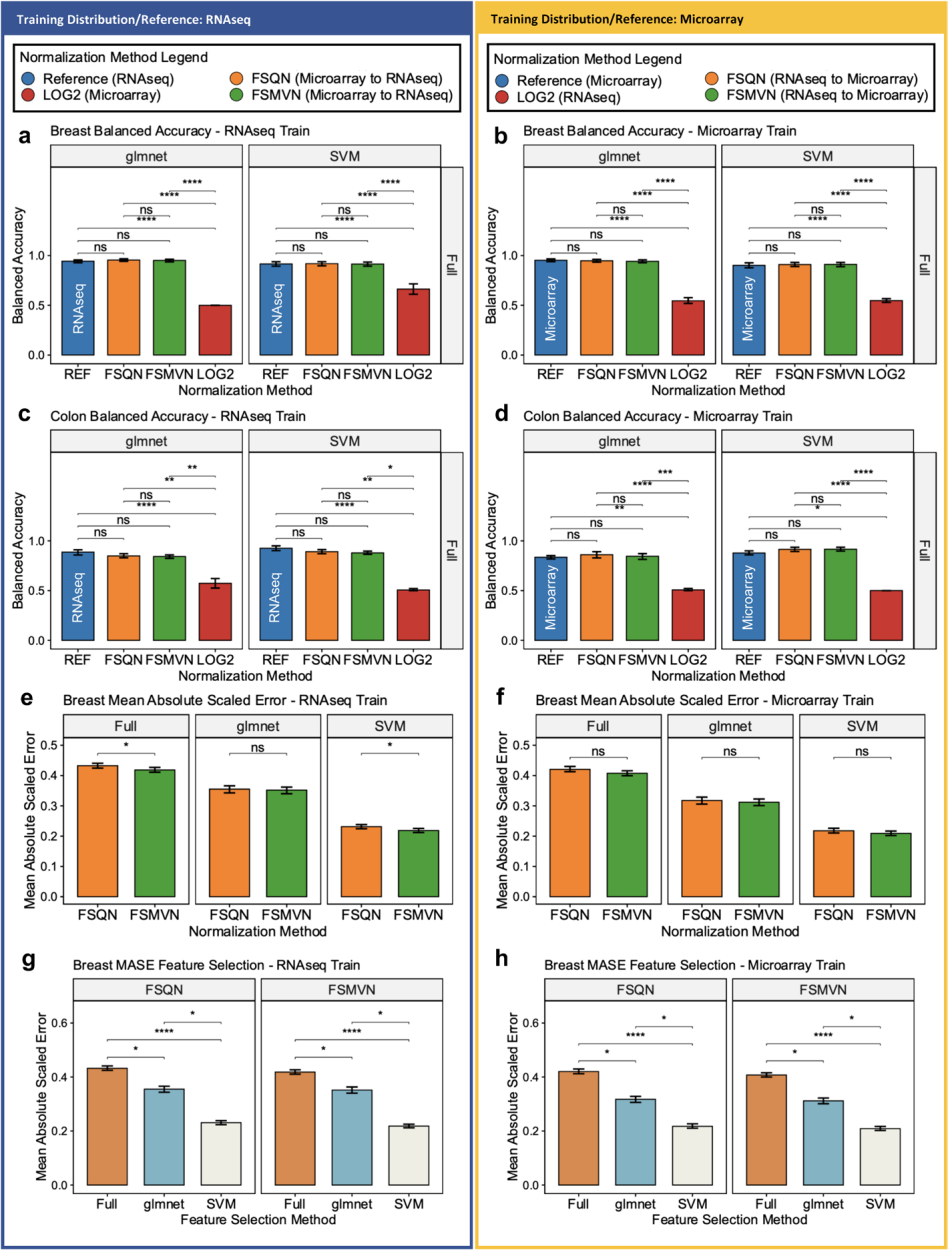
Model performance in PAM50 and CMS classification without feature selection. Left block (Dark Blue): Supervised classification using RNAseq data as training distribution. Right block (Gold): Supervised classification using microarray data as training distribution. Colour legends for each block are provided. All results are stratified by glmnet and SVM classification models. The y-axis label “Full” denotes models trained on all 12,638 genes (breast) or 13,362 genes (colon). **a**. Balanced accuracy (y-axis) derived from unseen out-of-fold test data from each normalization method (x-axis) for breast PAM50 classifier trained on RNAseq data. **b**. Balanced accuracy (y-axis) derived from unseen out-of-fold test data from each normalization method (x-axis) for breast PAM50 classifier trained on microarray data. **c**. Balanced accuracy (y-axis) derived from unseen out-of-fold test data from each normalization method (x-axis) for colon CMS classifier trained on RNAseq data. **d**. Balanced accuracy (y-axis) derived from unseen out-of-fold test data from each normalization method (x-axis) for colon CMS classifier trained on microarray data. 95% confidence intervals were calculated using 1,000 bootstraps with replacement. **e**. Mean absolute scaled error (y-axis) of breast gene expression data that is cross-platform normalized from microarray to RNAseq distribution for each normalization method (x-axis). **f**. Mean absolute scaled error (y-axis) of breast gene expression data that is cross-platform normalized from RNAseq to microarray distribution for each normalization method (x-axis). **g**. Mean absolute scaled error (y-axis) of breast gene expression data that is cross-platform normalized from microarray to RNAseq distribution according to feature selection method (x-axis) for FSQN and FSMVN, respectively. **h**. Mean absolute scaled error (y-axis) of breast gene expression data that is cross-platform normalized from RNAseq to microarray distribution according to each feature selection method (x-axis) for FSQN and FSMVN, respectively.. The significance of a Kruskal–Wallis with Dunn’s post-hoc test is annotated in the plot. (****p < 0.0001, ***p < 0.001, **p < 0.01, *p < 0.05, ns = not significant)

We evaluated the effect of FSQN and FSMVN on classification performance using “Full” models without feature selection (i.e., all genes were used to train models). In Fig. 3, we stratify the balanced accuracy by classification model. In all cases, balanced accuracy for PAM50 and CMS molecular classification was statistically equivalent between the reference training distribution and the experimental FSQN and FSMVN normalized distributions (Fig. 3). Balanced accuracy for the reference training distribution, FSQN, and FSMVN data was significantly greater than non-normalized log_2_ transformed data. Similar findings were reflected in the model’s Kappa performance metric (Additional file 1: Fig. S2).

### Cross-platform normalization and feature selection reduce mean absolute scaled error

We used MASE to evaluate the direct effects of FSQN and FSMVN on the gene expression values before and after cross-platform normalization. In some cases, FSMVN provided statistically significant reduction in MASE compared to FSQN (Fig. 3e and f). The absolute reduction in MASE between FSQN and FSMVN was minimal compared to log_2_ transformed data. For example, in breast models trained with RNAseq data, MASE in log_2_ transformation was 7.09 ± 0.13 (mean ± standard deviation) compared to 0.43 ± 0.01 and 0.42 ± 0.01 in FSQN and FSMVN, respectively. In Fig. 3g and h, we demonstrate that MASE is reduced with feature selection and that SVM provides a greater benefit compared to glmnet feature selection techniques in the breast cohort. Similar findings were identified in colon CMS (Additional file 1: Fig. S3a–d).

## FSQN and FSMVN are equally effective methods following feature selection

**Fig. 4:**
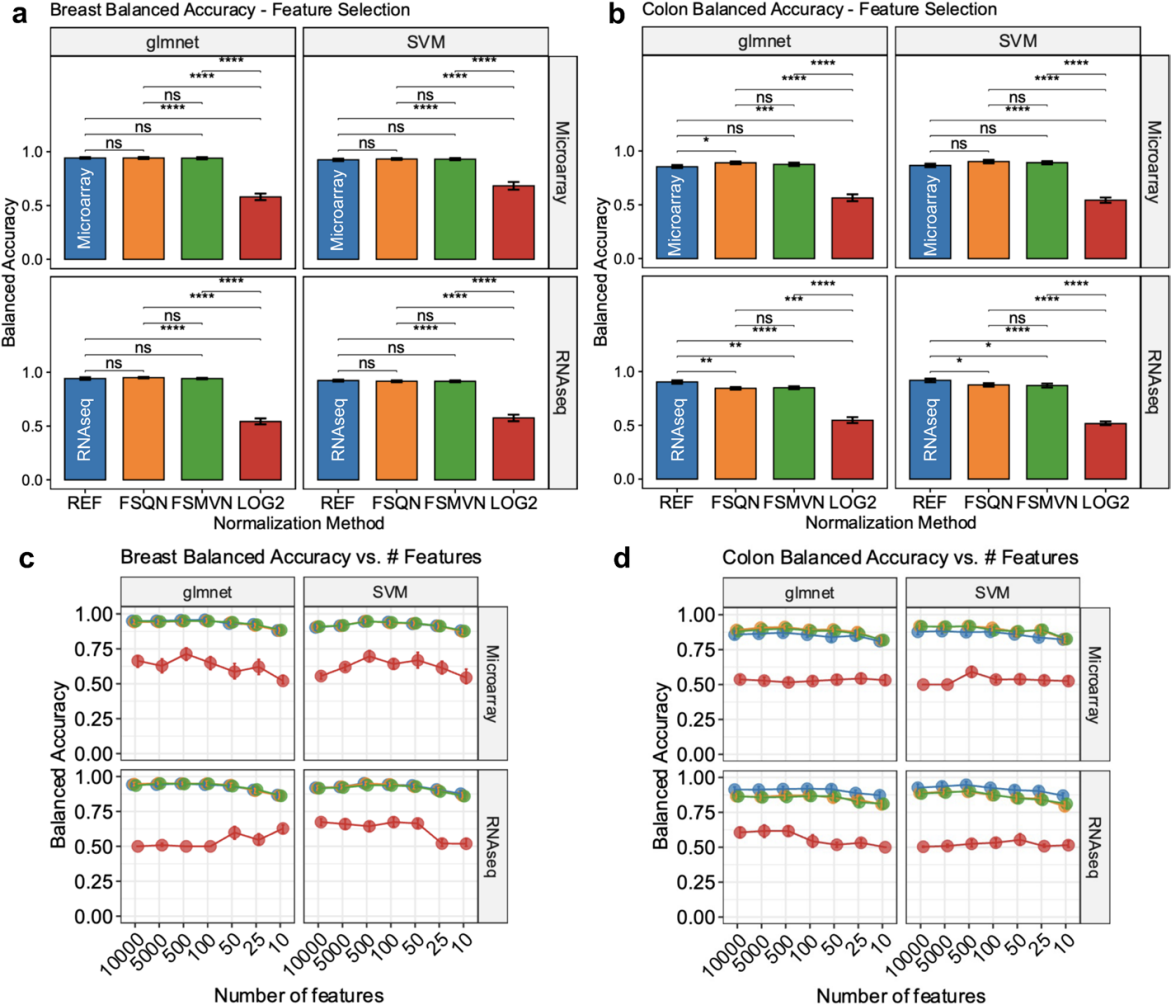
Model performance in PAM50 and CMS classification with feature selection. a. Balanced accuracy (y-axis) derived from unseen out-of-fold test data from each normalization method (x-axis) for breast PAM50 classifier using feature selection. **b**. Balanced accuracy (y-axis) derived from unseen out-of-fold test data from each normalization method (x-axis) for colon CMS classifier using feature selection. For **a** and **b**, the gray labels above the plot denote the feature selection method and the gray labels to the right denote the training distribution. **c**. Balanced accuracy (y-axis) derived from unseen out-of-fold test data versus the number of selected features (x-axis) for PAM50 classification. **d**. Balanced accuracy (y-axis) derived from unseen out-of-fold test data versus the number of selected features (x-axis) for CMS classification. For **c** and **d**, the gray labels above the plot denote the classifier model and the gray labels to the right denote the training distribution. Scatter plot colours correspond to the normalization method (blue = reference/training distribution, orange = FSQN, green = FSMVN, red = log_2_). 95% confidence intervals were calculated using 1000 bootstraps with replacement. The significance of a Kruskal–Wallis with Dunn’s post-hoc test is annotated in the plot. (****p < 0.0001, ***p < 0.001, **p < 0.01, *p < 0.05, ns = not significant)

Next, we assessed the effects of feature selection on cross-platform normalization methods. In Fig. 4a and b, we show the balanced accuracy of machine learning classifiers using an “optimal” feature selection procedure. In this scenario, features were selected using glmnet LASSO regression or SVM as described in the *Methods*.

For PAM50 classifiers, we found that performance was statistically equivalent between the reference training, FSQN, and FSMVN distributions (Fig. 4a). These findings were identified in models trained using RNAseq or microarray data and for features selected using glmnet or SVM for feature selection. Once again, the reference training, FSQN, and FSMVN distributions achieved significantly greater balanced accuracy compared to the non-normalized log_2_ distribution.

For colon CMS classification, FSQN and FSMVN achieved comparable balanced accuracy compared to the reference distribution (Additional file 1 and Fig. 4b). The reference distribution was found to have statistically superior performance compared to FSQN and FSMVN for models trained on RNAseq data with either glmnet or SVM feature selection (Fig. 4b). However, in models trained using microarray data, RNAseq-FSQN-to-microarray data achieved significantly greater balanced accuracy than the reference training distribution in the setting of glmnet feature selection (0.89 [95% CI 0.88–0.91] vs. 0.85 [95% CI 0.84–0.87]; p < 0.05). Similar findings were reflected in the model’s Kappa performance metric (Additional file 1: Fig. 4).

We performed multivariable regression to capture the effects of normalization methods, feature selection versus full modeling methods, and the training/testing distributions (Table 1). To compare normalization methods, we performed a post-hoc analysis of all pairwise comparisons of the marginal means. We found no significant difference in balanced accuracy between reference, FSQN, and FSMVN distributions (see Additional file 1). Using optimal feature selection techniques, we identified that feature selection methods were equivalent or significantly advantageous compared to “Full” model methods for PAM50 and CMS classification (Table 1). Overall, there were minimal clinically significant regression coefficients. For example, the effect of FSMVN relative to the reference distribution in Colon CMS classification was  − 1.3% despite achieving statistical significance. Beyond log_2_ normalization, regression coefficients only ranged from 0 to 3.4%.

**Table 1:** Balanced Accuracy of optimal multivariable regression models

	Breast			Colon		
Characteristic	Beta^a^	95% CI^b^	*p*-Value	Beta^a^	95% CI^b^	*p*-Value
Model						
glmnet	–	–		–	–	
SVM	− 0.001	− 0.009, 0.006	0.8	0.015	0.007, 0.023	< 0.001
Train distribution						
Agilent	–	–		–	–	
RNAseq	− 0.006	− 0.015, 0.002	0.15	0.016	0.007, 0.026	< 0.001
Test distribution						
Agilent	–	–		–	–	
RNAseq	0.006	− 0.002, 0.015	0.15	0.034	0.025, 0.044	< 0.001
Normalization method						
Reference (REF)	–	–		–	–	
FSQN	0.003	− 0.008, 0.014	0.6	− 0.006	− 0.017, 0.005	0.3
FSMVN	− 0.000	− 0.011, 0.011	> 0.9	− 0.013	− 0.024, − 0.001	0.031
LOG2	− 0.347	− 0.357, − 0.336	< 0.001	− 0.349	− 0.360, − 0.337	< 0.001
Feature selection method						
Full Model	–	–		–	–	
glmnet	0.009	0.001, 0.018	0.064	0.002	− 0.008, 0.012	0.7
SVM	0.012	0.002, 0.021	0.015	0.009	0.001, 0.019	0.083

^a^Beta = Percentage expressed as a decimal, ^b^*CI* Confidence Interval

We wanted to determine if the performance of FSQN and FSMVN is affected by the number of feature selected genes. In Fig. 4c we found that the reference training, FSQN, and FSMVN distributions have nearly identical performance in the PAM50 classification problem regardless of training distribution and number of features selected. For CMS classification the FSQN and FSMVN normalization tended to outperform the microarray training distribution. In contrast, the RNAseq training distribution achieved a marginally greater balanced accuracy compared to FSQN and FSMVN. In both cases, balanced accuracy was stable from 1,000 to 100 genes and began decreasing at 50 genes. Once again, similar findings were reflected in the model’s Kappa performance metric (Additional file 1: Fig. S4).

The effects of decreasing the number of feature selected genes in the context of other confounding variables were assessed using multivariable regression. In the PAM50 classification the FSQN and FSMVN were equivalent to the reference training distribution (Table 2). However, for CMS classification the reference distribution was found to achieve statistically greater balanced accuracy compared to FSQN and FSMVN for CMS classification. This result was arguably not clinically significant given that FSQN and FSMVN were estimated to contribute 1.0% and 1.3% less accuracy relative to the reference distribution. Pairwise comparisons of the normalization methods maintained the finding of greater balanced accuracy in the reference distribution compared to FSQN and FSMVN for CMS classification (Ref > FSQN, *p* < 0.05; Ref > FSMVN, *p* < 0.01) (Additional file 1).

**Table 2:** Balanced accuracy number of selected features regression models

	Breast			Colon		
Characteristic	Beta^a^	95% CI^b^	*p*-Value	Beta^a^	95% CI^b^	*p*-Value
*Number of features*						
10,000	–	–		–	–	
5000	0.006	− 0.005, 0.016	0.3	0.003	− 0.007, 0.013	0.5
500	0.025	0.014, 0.036	< 0.001	0.013	0.003, 0.023	0.012
100	0.017	0.006, 0.028	0.002	− 0.003	− 0.014, 0.007	0.5
50	0.012	0.001, 0.022	0.033	− 0.012	− 0.023, − 0.002	0.015
25	− 0.020	− 0.030, − 0.009	< 0.001	− 0.023	− 0.033, − 0.013	< 0.001
10	− 0.053	− 0.064, − 0.042	< 0.001	− 0.054	− 0.064, − 0.044	< 0.001
*Model*						
glmnet	–	–		–	–	
SVM	0.001	− 0.005, 0.006	0.8	0.004	− 0.001, 0.010	0.12
*Train distribution*						
Agilent	–	–		–	–	
RNAseq	− 0.010	− 0.016, − 0.003	0.003	0.020	0.013, 0.026	< 0.001
*Test distribution*						
Agilent	–	–		–	–	
RNAseq	0.007	0.000, 0.014	0.038	0.035	0.029, 0.041	< 0.001
*Normalization method*						
Reference (REF)	–	–		–	–	
FSQN	− 0.002	− 0.010, 0.006	0.7	− 0.010	− 0.018, − 0.002	0.010
FSMVN	− 0.002	− 0.011, 0.006	0.6	− 0.013	− 0.021, − 0.006	< 0.001
LOG2	− 0.324	− 0.332, − 0.316	< 0.001	− 0.347	− 0.355, − 0.340	< 0.001

^a^Beta Percentage expressed as a decimal, ^b^*CI* Confidence Interval

## Discussion

Accurate cross-platform normalization allows integration of whole transcriptome gene-expression data. Reliable normalization from RNA-sequencing platforms to microarray and vice-versa provides researchers with a tool to test hypotheses on external datasets, which aids in replication and validation of findings. Furthermore, these methods may also allow translational delivery of findings derived from costly whole transcriptome data to affordable, and efficient tests such as Nanostring.

For the purposes of using cross-platform normalization in supervised machine learning models, we establish that FSQN and FSMVN are equivalent in terms of balanced accuracy and Kappa metrics. In the case of breast PAM50 classification, FSQN and FSMVN delivered statistically equivalent model performance on cross-platform normalization data compared to within-platform data. This was also true for colon CMS classification in the context of no feature selection and SVM models with feature selection. Overall, FSQN and FSMVN provided significant improvement in MASE of the actual gene expression values, but these significant differences were not translated to the primary outcome of balanced accuracy. Moreover, we provide the first validation of a commonly used, but previously unnamed method we label as Feature Specific Mean Variance Normalization.

Previous work has established the utility of FSQN compared to other prominent cross-platform normalization methods. In this study, we provide novel results supporting the utility of FSQN and FSMVN in normalizing gene-expression data in a bidirectional manner (i.e., microarray to RNAseq and vice versa). Furthermore, our study provides the first unbiased assessment of FSQN and FSMVN in the context of feature selection by using nested cross-validation methods.

We identified that model performance differences may exist depending on the classification problem, the machine learning model used, or the training and testing distribution. For example, in the colon CMS classification model performance was model dependent on the training distribution and the feature selection model used. Based on this information we recommend that researchers continue to approach cross-platform normalization with caution. We encourage researchers to use a variety of models to identify the optimal combination. If possible, we also encourage external validation of classification models using alternative biological or clinical outcomes.

These methods are not without limitations. FSQN and FSMVN require matching distributions with identical genes. Depending on the specific technology, tissue or probe set used, it is not uncommon to have discordance between the measured genes. Thus, these methods suffer where missing gene-level data occurs between separate platforms. Potential avenues to remedy this problem include missing data imputation methods such as k-nearest neighbour imputation or multivariate imputation by chained equations [31, 32]. Another limitation is that we only examined distributions within the same cancer type. Normalization in the context of multi-cancer or pan-cancer applications may be affected by distributional discrepancies among unique tissues. Further investigation of these limitations is required in future studies.

## Conclusions

In this study, we demonstrate that FSQN and FSMVN are effective methods for cross-platform normalization. These methods allow the normalization of microarray data to RNAseq data and vice-versa. The validity of previous research using FSMVN is augmented by these results. To aid in the replication of these methods we have provided an R function for FSMVN. Finally, these methods are valid in the context of feature selection. Future study includes evaluating the validity of cross-platform normalization to perform pooled differential gene expression analysis and assessment of missing gene value techniques.

### Supplementary Information

**Additional file 1: **Feature Specific Quantile Normalization R Function **Supplementary Figure 1.** Effect of feature specific normalization methods on test and training colon cancer distributions. **Supplementary Figure 2.** Model Kappa performance in PAM50 and CMS classification without feature selection. **Supplementary Figure 3.** Model performance according to Mean Absolute Scaled Error for Colon CMS gene expression data. **Supplementary Figure 4.** Model performance in PAM50 and CMS classifications with feature selection.

## Data Availability

Publicly available RNAseq and microarray data were retrieved from cbioportal or Genomic Data Commons. Phenotype data was retrieved from cbioportal for TCGA BRCA and from Synapse (ID syn2623706) for TCGA COAD CMS subtypes. [19, 22] Software package for Feature Specific Quantile Normalization is available from the ‘FSQN’ package (https://github.com/jenniferfranks/FSQN). Code for the Feature Specific Mean Variance Normalization function in R is available in the Additional file 1.
