# Supplementary material for: Feature-specific quantile normalization and feature-specific mean–variance normalization deliver robust bi-directional classification and feature selection performance between microarray and RNAseq data
Source: BMC Bioinformatics. 2024 Mar 29;25:136. doi: 10.1186/s12859-024-05759-w (PMC11265146; doi:10.1186/s12859-024-05759-w)
Supplement: Supplementary file 1 — Additional file 1: Feature Specific Quantile Normalization R Function Supplementary Figure 1. Effect of feature specific normalization methods on test and training colon cancer distributions. Supplementary Figure 2. Model Kappa performance in PAM50 and CMS classification without feature selection. Supplementary Figure 3. Model performance according to Mean Absolute Scaled Error for Colon CMS gene expression data. Supplementary Figure 4. Model performance in PAM50 and CMS classifications with feature selection. [file 12859_2024_5759_MOESM1_ESM.docx]

**Supplementary Material**

Contents:

1. Feature Specific Quantile Normalization R Function
2. Supplementary Figure 1. Effect of feature specific normalization methods on test and training colon cancer distributions.
3. Supplementary Figure 2. Model Kappa performance in PAM50 and CMS classification without feature selection.
4. Supplementary Figure 3. Model performance according to Mean Absolute Scaled Error for Colon CMS gene expression data.
5. Supplementary Figure 4. Model performance in PAM50 and CMS classifications with feature selection.

Feature Specific Quantile Normalization R Function

FSMVN <- function(test, target) {

xcenter = colMeans(test)

sd_byfeature = apply(test,2,sd)

sd_byfeature_tar = apply(target,2,sd)

xcenter_tar = colMeans(target)

(((test - rep(xcenter, rep.int(nrow(test), ncol(test))))/rep(sd_byfeature, rep.int(nrow(test), ncol(test))))* rep(sd_byfeature_tar, rep.int(nrow(test), ncol(test)))) + rep(xcenter_tar, rep.int(nrow(test), ncol(test)))

}


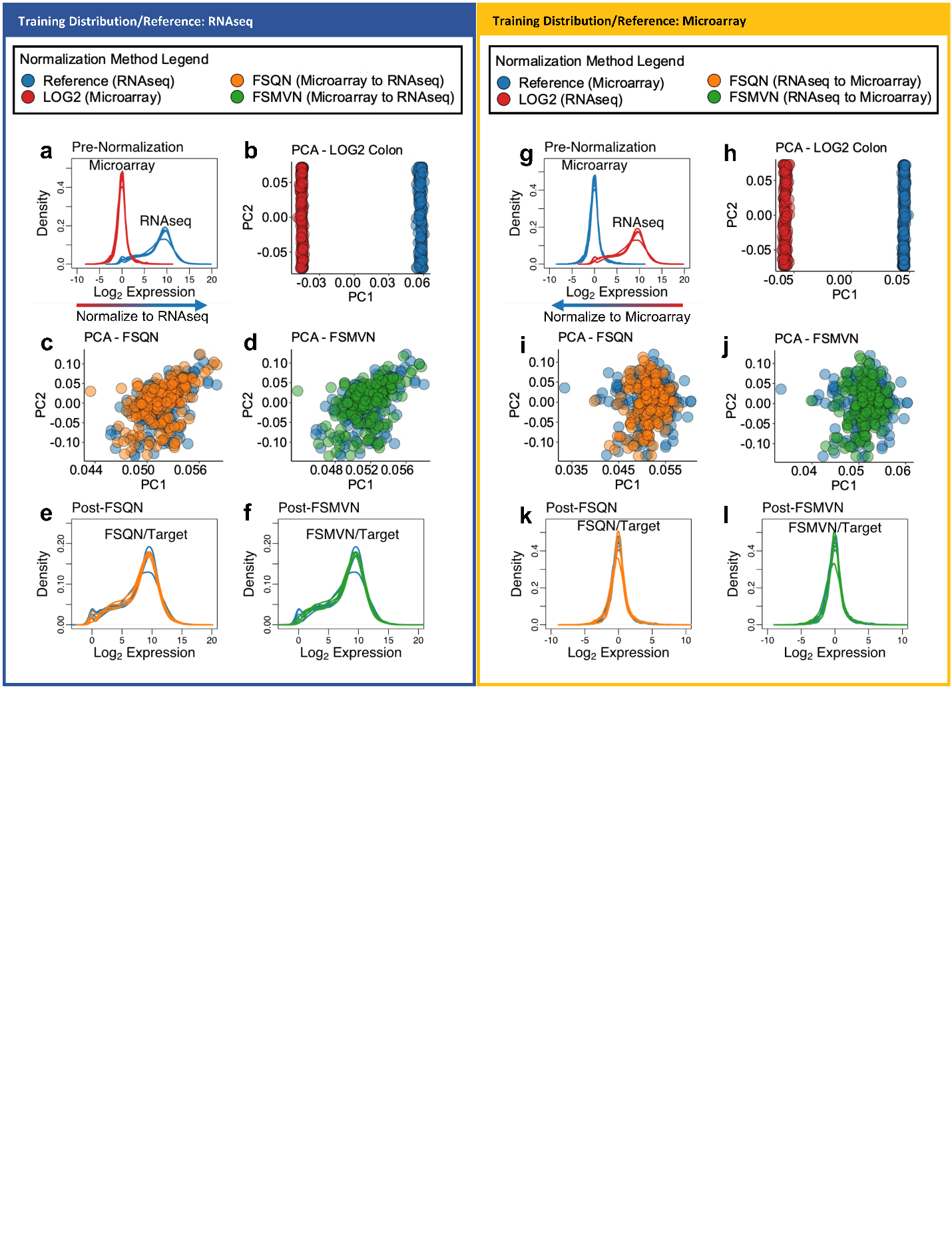


**Supplementary Figure 1.** Effect of feature specific normalization methods on test and training colon cancer distributions. Left block (Dark Blue): Normalization using RNAseq data as training distribution. Right block (Gold): Normalization using microarray data as training distribution. Colour legends for each block are provided. **a, g**. Probability density functions of log_2_ microarray and RNAseq data prior to feature specific normalization. **b, h**. Principal Component Analysis (PCA) plots of log_2_ microarray data and log_2_ RNAseq data. The first (PC1) and second (PC2) principal components are projected on the x-axis and y-axis, respectively. **c, i.** PCA plot of the first two principal components of gene expression data after feature specific quantile normalization (orange) to the respective training distribution (blue) demonstrates limited variation between gene expression platforms after FSQN. **d, j**. PCA plot of the first two principal components of gene expression data after feature specific mean-variance normalization (green) to training distribution (blue) demonstrates limited variation between gene expression platforms after FSMVN. **e, k**. The probability density function of gene expression data after FSQN demonstrates the shift of the test distribution (orange) to match the training distribution (blue). **f, l**. The probability density function of gene expression data after FSMVN demonstrates the shift of the test distribution (green) to match the training distribution (blue).


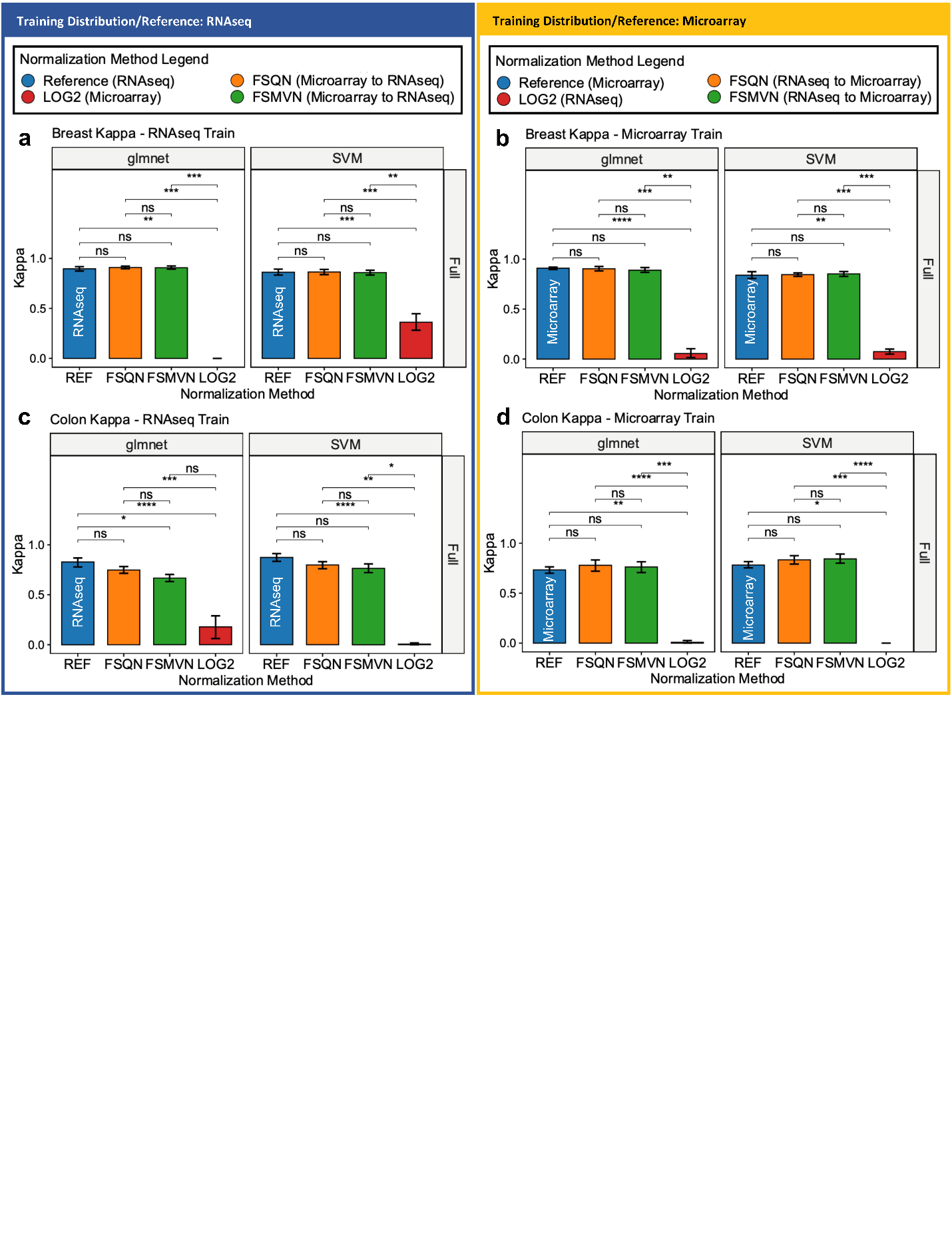


**Supplementary Figure 2.** Model Kappa performance in PAM50 and CMS classification without feature selection. Left block (Dark Blue): Supervised classification using RNAseq data as training distribution. Right block (Gold): Supervised classification using microarray data as training distribution. Colour legends for each block are provided. All results are stratified by glmnet and SVM classification models. The y-axis label “Full” denotes models trained on all 12,638 genes (breast) or 13,362 genes (colon). **a**. Kappa (y-axis) derived from unseen out-of-fold test data from each normalization method (x-axis) for breast PAM50 classifier trained on RNAseq data. **b**. Kappa (y-axis) derived from unseen out-of-fold test data from each normalization method (x-axis) for the breast PAM50 classifier trained on microarray data. **c**. Kappa (y-axis) derived from unseen out-of-fold test data from each normalization method (x-axis) for colon CMS classifier trained on RNAseq data. **d**. Kappa (y-axis) derived from unseen out-of-fold test data from each normalization method (x-axis) for colon CMS classifier trained on microarray data. 95% confidence intervals were calculated using 1,000 bootstraps with replacement. The significance of a Kruskal-Wallis with Dunn’s post-hoc test is annotated in the plot. (****p<0.0001, ***p<0.001, **p<0.01, *p<0.05, ns = not significant).

**
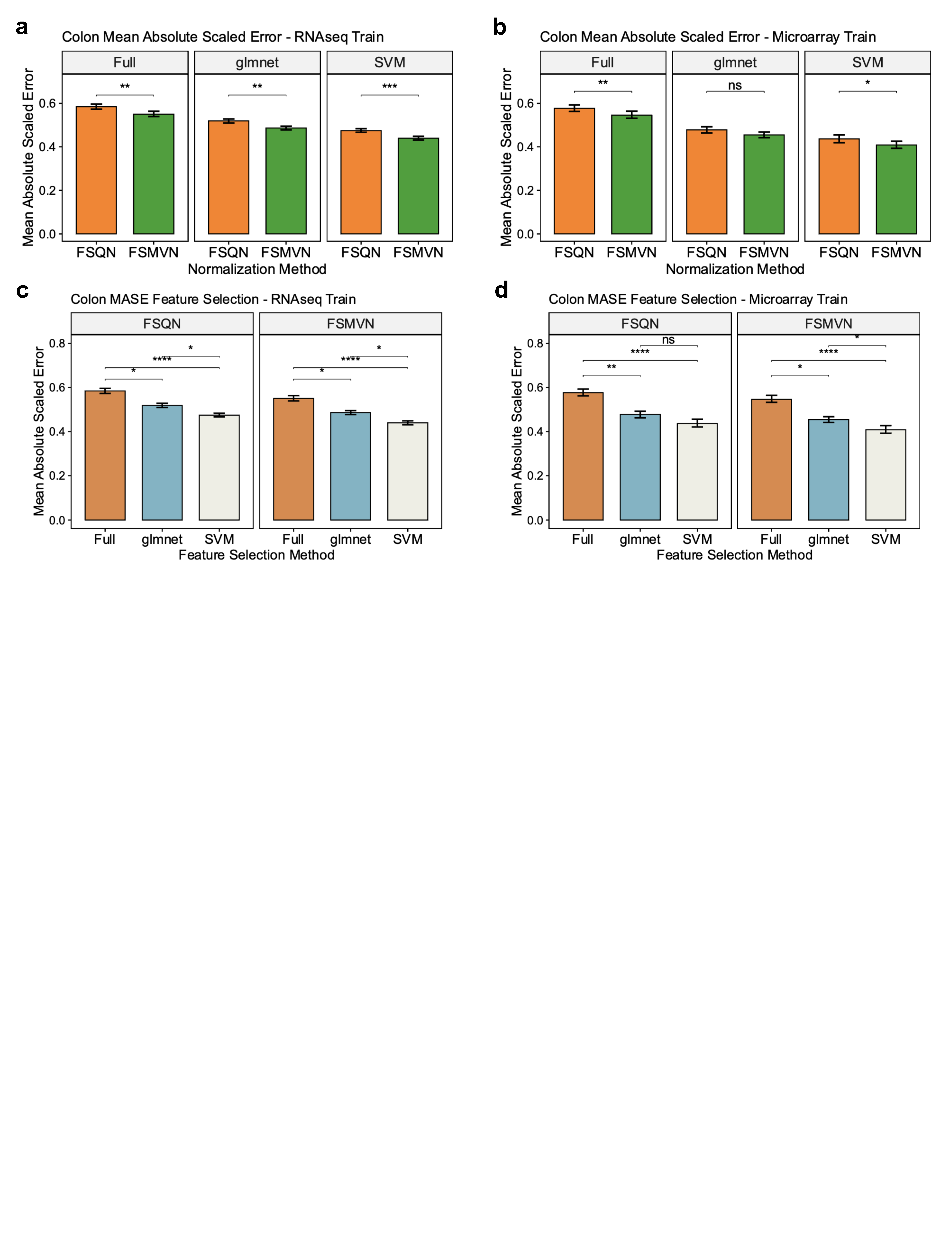
**

**Supplementary Figure 3.** Model performance according to Mean Absolute Scaled Error for Colon CMS gene expression data. **a.** Mean absolute scaled error (y-axis) of colon gene expression data that is cross-platform normalized from microarray to RNAseq distribution for each normalization method (x-axis). **b.** Mean absolute scaled error (y-axis) of colon gene expression data that is cross-platform normalized from RNAseq to microarray distribution for each normalization method (x-axis). **c.** Mean absolute scaled error (y-axis) of colon gene expression data that is cross-platform normalized from microarray to RNAseq distribution according to feature selection method (x-axis) for FSQN and FSMVN, respectively. **d.** Mean absolute scaled error (y-axis) of colon gene expression data that is cross-platform normalized from RNAseq to microarray distribution according to each feature selection method (x-axis) for FSQN and FSMVN, respectively.. The significance of a Kruskal-Wallis with Dunn’s post-hoc test is annotated in the plot. (****p<0.0001, ***p<0.001, **p<0.01, *p<0.05, ns = not significant).


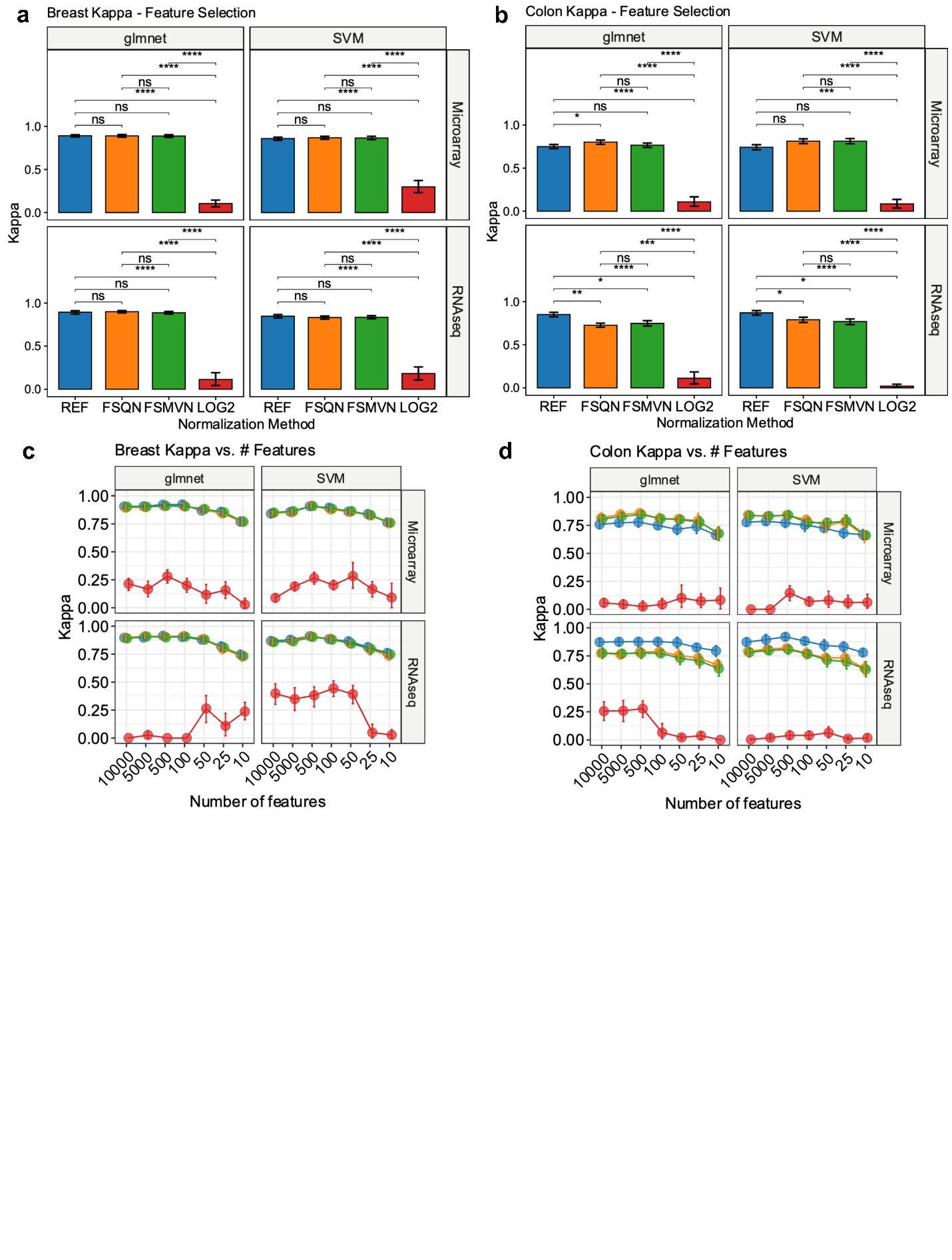


**Supplementary Figure 4.** Model performance in PAM50 and CMS classifications with feature selection. **a**. Kappa (y-axis) derived from unseen out-of-fold test data from each normalization method (x-axis) for breast PAM50 classifier using feature selection. **b**. Kappa (y-axis) derived from unseen out-of-fold test data from each normalization method (x-axis) for colon CMS classifier using feature selection. For **a** and **b**, the gray labels above the plot denote the feature selection method and the gray labels to the right denote the training distribution. **c**. Kappa (y-axis) derived from unseen out-of-fold test data versus the number of selected features (x-axis) for PAM50 classification. **d**. Kappa (y-axis) derived from unseen out-of-fold test data versus the number of selected features (x-axis) for CMS classification. For **c** and **d**, the gray labels above the plot denote the classifier model and the gray labels to the right denote the training distribution. Scatter plot colours correspond to the normalization method (blue = reference/training distribution, orange = FSQN, green = FSMVN, red = log_2_). 95% confidence intervals were calculated using 1,000 bootstraps with replacement. The significance of a Kruskal-Wallis with Dunn’s post-hoc test is annotated in the plot. (****p<0.0001, ***p<0.001, **p<0.01, *p<0.05, ns = not significant).
